# Interrelationships of VEL1 and ENV1 in light response and development in *Trichoderma reesei*

**DOI:** 10.1371/journal.pone.0175946

**Published:** 2017-04-19

**Authors:** Hoda Bazafkan, Christoph Dattenböck, Eva Stappler, Sabrina Beier, Monika Schmoll

**Affiliations:** AIT Austrian Institute of Technology GmbH, Department Health and Environment, Bioresources, Tulln, Austria; Georg-August-University of Göttingen Institute of Microbiology & Genetics, GERMANY

## Abstract

Sexual development is regulated by a complex regulatory mechanism in fungi. For *Trichoderma reesei*, the light response pathway was shown to impact sexual development, particularly through the photoreceptor ENVOY. Moreover, *T*. *reesei* communicates chemically with a potential mating partner in its vicinity, a response which is mediated by the velvet family protein VEL1 and its impact on secondary metabolism. We therefore studied the regulatory interactions of ENV1 and VEL1 with a focus on sexual development. Although individual mutants in both genes are female sterile under standard crossing conditions (light—dark cycles), an altered light regime enabled sexual development, which we found to be due to conditional female sterility of Δ*env1*, but not Δ*vel1*. Phenotypes of growth and asexual sporulation as well as regulation of the peptide pheromone precursors of double mutants suggested that ENV1 and VEL1 balance positive and negative regulators of these functions. Additionally, VEL1 contributed to the strong deregulation of the pheromone system observed in *env1* mutants. Female sterility of Δ*vel1* was rescued by deletion of *env1* in darkness in MAT1-1, indicating a block of sexual development by ENV1 in darkness that is balanced by VEL1 in the wild-type. We conclude that ENV1 and VEL1 exert complementing functions in development of *T*. *reesei*. Our results further showed that the different developmental phenotypes of *vel1/veA* mutants in *T*. *reesei* and *Aspergillus nidulans* are not due to the presence or function of ENV1 in the VELVET regulatory pathway in *T*. *reesei*.

## Introduction

*Trichoderma reesei* is predominantly known as a biotechnological workhorse for production of plant cell wall degrading enzymes and heterologous proteins, which are regulated in response to different carbon sources, nutrient sources and light [1–3]. Sexual development under laboratory conditions has been achieved in *T*. *reesei* only a few years ago [4, 5]. *T*. *reesei* has two pheromone receptors (HPR1 and HPR2) as well as two peptide pheromone precursor genes (*hpp1* and *ppg1*), where *hpp1* represents a novel class of peptide pheromone precursors, but assumes a-type function [6, 7]. For mating, a pair of receptor and cognate pheromone precursor, i. e. *ppg1* and *hpr2* or *hpp1* and *hpr1* is required. Moreover, as in other fungi, lack of the mating type associated pheromone receptor leads to female sterility and deletion of the pheromone precursors leads to male sterility [7, 8].

In fungi, sexual development is influenced by diverse environmental factors, including temperature and nutrient availability. In most species, light plays an important role for the decision whether to reproduce sexually or asexually [9, 10]. *T*. *reesei* initiates sexual development predominantly upon growth in light, with components of the light response pathway being involved in regulation of mating [11].

The photoreceptors BLR1 and BLR2 (blue light regulator 1 and 2) [12] were found to influence the pheromone system as well as fruiting body formation, but they are not essential for mating [11, 13]. A much stronger effect was found for the third photoreceptor, ENV1 (Envoy1). ENV1 is crucial for proper regulation of the pheromone system, which becomes de-regulated in light upon deletion of *env1*. This deregulation causes female sterility which is assumed to be due to loss of sexual identity [11]. Moreover, ENV1 is responsible for dampening the inhibitory effect of constant light on sexual development in response to changes in illumination [13]. ENV1 is a PAS/LOV domain protein and represents an orthologue to the photoreceptor VVD of *N*. *crassa* [14–16]. In *N*. *crassa*, VVD acts as a universal brake to photoresponses [17, 18] and serves as a molecular memory for the brightness of the preceding day, which enables *N*. *crassa* to distinguish between daylight and moonlight [19]. ENV1 is assumed to exert its function via modification of the activity of the BLR1/BLR2 photoreceptor complex, both of which are transcription factors [12, 16]. Nevertheless, ENV1 also impacts gene regulation independent of BLR1/BLR2 [20] and acts at least in part via modulation of the cAMP pathway [21]. ENV1 predominantly regulates gene expression in light but also has functions in darkness. However, overexpression of ENV1 in darkness is not sufficient to exert light-state functions, hence indicating the contribution of additional components [22]. Additionally, ENV1 was found to integrate responses to oxidative and osmotic stress in light via distinct, evolutionarily conserved amino acids [23, 24].

*A*. *nidulans* VeA (Velvet A) activates sexual development and inhibits asexual development [25, 26]. The Velvet family of regulatory proteins exerts key functions in coordination of secondary metabolism and developmental and differentiation processes [27]. In *A*. *nidulans*, sexual development is favored in darkness, while asexual development is preferred in light [28], which is in contrast to the situation in *T*. *reesei* [5]. VeA is a light dependent regulator of sexual development and asexual sporulation acting through a mechanism that involves interaction with the phytochrome FphA, nuclear-cytoplasmatic shuttling and complex formation with photoreceptors [29–31]. Importantly, *A*. *nidulans* does not possess an ENV1 homologue [32]. Consequently, differences in function and relevance of light dependent regulators of development that are responsible for the phenotypic differences in sexual and asexual development between *Aspergilli* [33] and *T*. *reesei* [34] have to be expected in these two fungi.

The differences in developmental functions of the photoreceptors LreA and LreB (homologues of BLR1 and BLR2) between *T*. *reesei* and *A*. *nidulans* are reflected in considerable defects in cleistothecium formation in Δ*lreA* and Δ*lreB* mutants in light and darkness [30], while in *T*. *reesei* only minor effects of deletion of *blr1* or *blr2* or both were observed [11].

For *T*. *reesei* previous data showed that the VeA homologue VEL1 serves as a molecular link between light signaling, development and secondary metabolism [34]. Thereby it functions in partner sensing and chemical communication between potential mating partners. VEL1 is essential for sexual development in darkness and for female fertility in light and regulates transcript levels of the pheromone system genes, in part also depending on partner sensing [34]. Lack of VEL1 causes abolishment of conidiation in *T*. *reesei* [34] and *T*. *virens* [35]. Further functions of VEL1 in *T*. *reesei* include regulation of cellulase gene expression [36] and in *T*. *virens* VEL1 is important for biocontrol, mycoparasitism and morphology as well as secondary metabolism [35] as also shown in *T*. *reesei*. The relevance of ENV1 on regulatory mechanisms involving VEL1 has not yet been studied in any fungus.

Here we show a regulatory interrelationship between the photoreceptor ENV1 and VEL1, an important regulator of secondary metabolism and sexual development. ENV1 and VEL1 show interdependent functions in regulation of growth, sporulation, pheromone response and sexual development.

## Results

### *Vel1* is regulated by ENV1 in light

**Fig 1:**
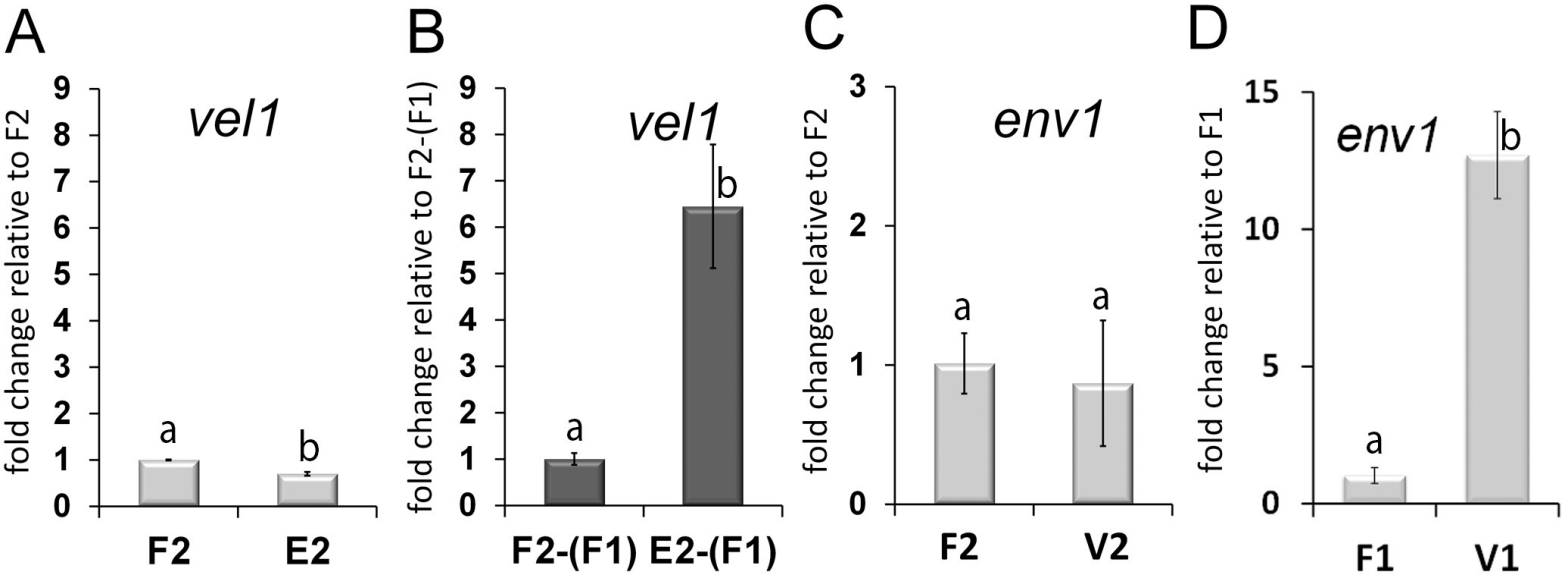
Analysis of mutual regulation of ENV1 and VEL1. Transcript abundance is shown upon growth on malt extract agar plates at subjective noon as analyzed by qRT-PCR in the female fertile wildtype strain FF1 (“F1”; MAT1-1), (FF2 (“F2”; MAT1-2), in Δ*env1F2* (“E2”; MAT1-2) or in Δ*vel1F2* (“V2”; MAT1-2). (A) Regulation of *vel1* transcript abundance by wildtype and in a strain lacking *env1* under asexual conditions. (B) Regulation of *vel1* transcript abundance under sexual conditions in wildtype F2-(F1) and Δ*env1F2* E2-(F1) upon encounter of the female fertile wildtype strain FF1 (“F1”; MAT1-1). (C) Regulation of *env1* transcript abundance by VEL1 under asexual conditions in MAT1-2 and (D) in MAT1-1. Pooled samples from several plates and at least two independent biological replicates were considered. Statistical significance of differences was evaluated with the software qbase+ and applying ANOVA analysis with a p-value threshold of p<0.05. Errorbars show standard deviations. Different letters indicate significant differences. For investigation of sexual development, contamination of total RNA of one sample with that of the respective mating partner on the same plate was determined to be below 1.02%.

Screening of available transcriptome data for *T*. *reesei* indicated only small variations in transcript abundance of *vel1* upon growth on cellulose in light or darkness and upon lack of the heterotrimeric G-protein components GNB1 (G-protein beta subunit), GNG1 (G-protein gamma subunit) or PhLP1 (phosducin) [37]. However, evaluating targets of photoreceptors, ENV1 showed a clearly negative effect (roughly 2fold) on transcript levels of *vel1* upon growth on cellulose in light [20], an effect which was not described for other ENV1/VVD homologues before. Transcript levels of *vel1* in Δ*env1F*, a strain lacking *env1* in a female fertile background [34], were assessed by qRT-PCR. During asexual growth on malt extract agar plates we detected a minor decrease in transcript abundance of *vel1* by 30 ± 4% compared to wildtype (Fig 1A), but in the presence of a mating partner, *vel1* levels increased more than 6 fold (6.45 ± 1.33 fold) in Δ*env1* compared to wildtype under mating conditions (Fig 1B).

Due to the effect of ENV1 on *vel1* transcription we wanted to analyze the position of these two factors in the signaling cascade by qRT-PCR. Transcript levels of *env1* were not significantly influenced by VEL1 upon growth on malt extract media in MAT1-2 (Fig 1C), but negatively regulated in MAT1-1 (Fig 1D). No significant regulation was observed under sexual conditions.

### Altered light conditions enable mating between mutants in *env1* and *vel1*

**Fig 2:**
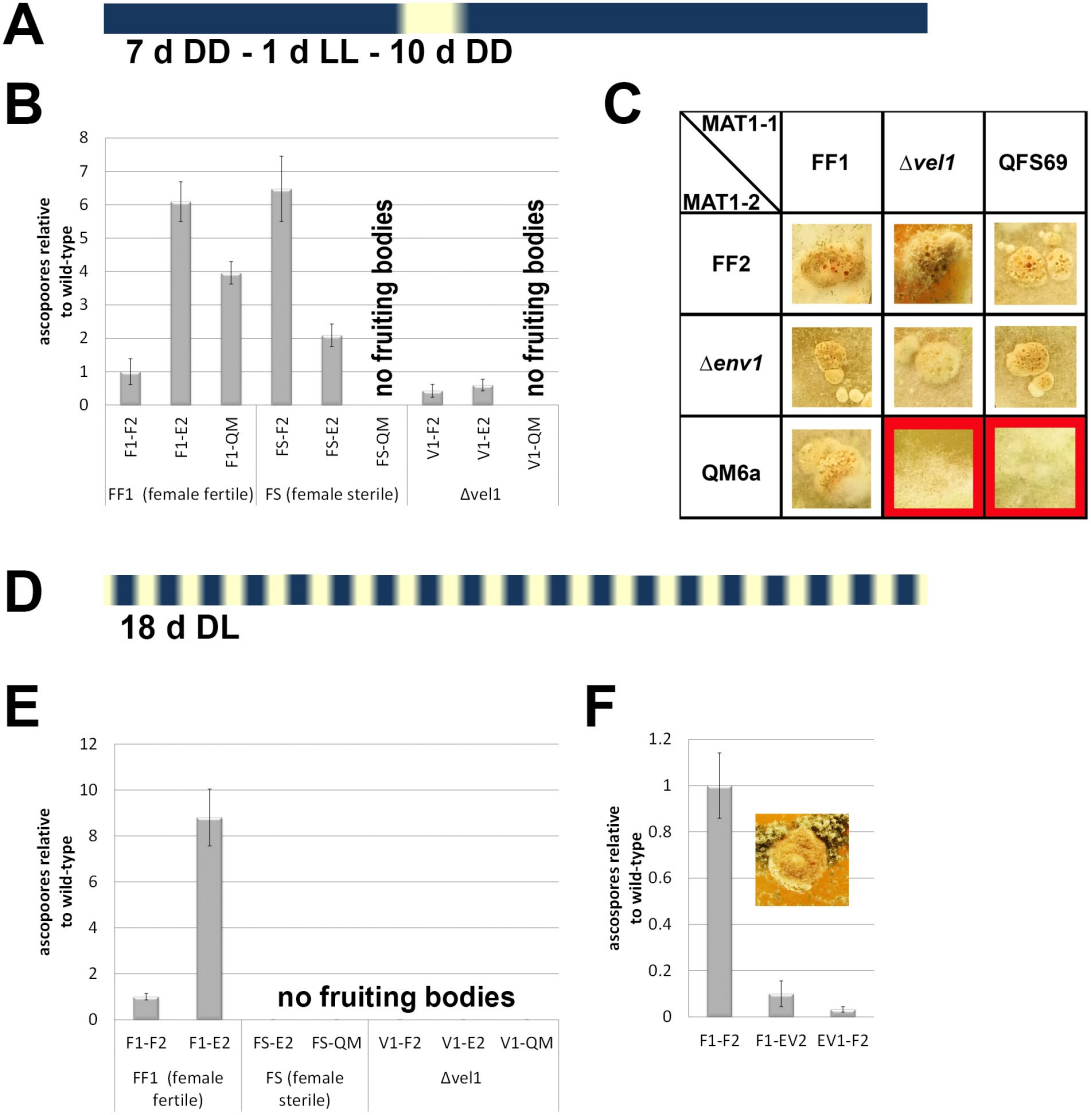
Fruiting body formation and ascospore discharge under altered light conditions. (A) Schematic representation of the altered light regime to enable mating of Δ*env1*F and Δ*vel1*F. (B) Ascospore discharge after 18 days of cultivation under the altered light regime, normalized to the wildtype cross (F1-F2). (C) Fruiting body formation under the altered light conditions. Representative fruiting bodies from 10 replicates cultivated under similar conditions are shown. Red background indicates no fruiting body formation. (D) Schematic representation of light conditions for control cultures under conventional conditions for crossing (daylight, 12:12 cycles). (E) Ascospore discharge after 18 days of cultivation under conventional light-dark cycles, normalized to the wildtype cross (F1-F2). (F) Ascospore discharge of Δ*env1*Δ*vel1*F double mutants in both mating types after 18 days (light-dark cycles), relative to the wild-type cross (F1-F2). The insert shows a typical fruiting body of a cross of double mutants with wild-type. As Δ*env1*Δ*vel1*F double mutants are female sterile, they were not able to undergo sexual development with each other. Strains were grown on malt extract medium (3% w/v) at 22°C to enable mating for 18 days under the light conditions described above. Five biological replicates were considered for the analysis and errorbars show standard deviations. Abbreviations: F1: strain FF1, MAT1-1, and F2: strain FF2, MAT1-2 are female fertile derivatives of QM6a obtained by backcrossing [34]; QM: QM6a (MAT1-2); FS: QFS69 (MAT1-1) female sterile derivative of QM6a, sister strain of FF1 and FF2 [34] E2: Δ*env1*F MAT1-2; V1: Δ*vel1*F MAT1-1; EV1 and EV2: Δ*env1*Δ*vel1*F double mutants in both mating types.

It is assumed that the strongly up-regulated pheromone system in strains lacking ENV1 hampers proper mate recognition, which causes the observed female sterility of Δ*env1* in light [11]. However, upon growth in darkness, deletion of *env1* does not result in altered transcript patterns of pheromone receptor (*hpr1*, *hpr2*) or pheromone precursor genes (*hpp1*, *ppg1*) under sexual conditions, and fruiting body formation occurs [11]. In contrast, *vel1* is essential for fruiting body formation in darkness and crossing under usually applied conditions, or in constant light or in constant darkness was not possible. Consequently, we hypothesized that strains lacking *env1* and *vel1* as partners might be able to undergo sexual development under altered combinations of light-dark periods. To test this, crosses were performed between Δ*vel1F* and Δ*env1F* in both mating types under diverse light regimes to enable mating between these strains. The successful combination was as follows: Crosses were first incubated in constant darkness for 7 days, in order to avoid deregulation of the pheromone system of Δ*env1* in the initial phase. Thereafter the crosses were incubated in light for one day in order to provide light pulses for Δ*vel1F* strains to initiate sexual development. Finally, the plates were kept in constant darkness for 10 days again to limit overproduction of pheromones by Δ*env1F* (Fig 2A). Crosses treated with this light regime resulted in fruiting bodies with pale brown color similar to wildtype fruiting bodies in constant darkness and ascospore discharge occurred (Fig 2B and 2C). Ascospore discharge of these fruiting bodies was however very low (Fig 2B), but still sufficient for isolation of double mutants in *env1* and *vel1*. Hence, the defect of Δ*vel1F* and/or Δ*env1F* in female fertility is condition dependent.

### Female sterility of Δ*env1*, but not Δ*vel1* is condition dependent

To further understand the mechanism of the fruiting body formation between Δ*env1* and Δ*vel1* and to be able to explain the observed phenomenon, we applied the light regime that previously resulted in mating between Δ*env1F* and Δ*vel1F* to additional crosses with female sterile strains of opposite mating type (QM6a MAT 1–2 or QFS69 MAT 1–1, a female sterile derivative of QM6a). Crosses between Δ*env1F* and Δ*vel1F* as described above served as controls.

Crosses between the female sterile strains and Δ*vel1F* were not successful under the special light regime (Fig 2C). Therefore, female sterility of Δ*vel1F* is consistent and confirmed also under the altered light condition, while female sterility of Δ*env1F* turned out to be light-condition dependent, which is in accordance with the influence of ENV1 on the pheromone system in light, but not in darkness.

Analysis of ascospore discharge further showed that under the altered light conditions, crosses with a female sterile mating partner (QM6a or QFS69), but also with the conditional female sterile Δ*env1F* resulted in higher ascospore levels than a wild-type cross (Fig 2B). Comparative analysis under 12:12 light—dark cycles (Fig 2D) showed that also under these conditions a cross with Δ*env1F* yields higher amounts of ascospores (Fig 2E). Ascospore discharge of Δ*env1*Δ*vel1F* double mutants is strongly decreased to less than 10% of wild-type crosses (Fig 2F).

### ENV1 and VEL1 have interrelated functions in regulation of growth and sporulation

**Fig 3:**
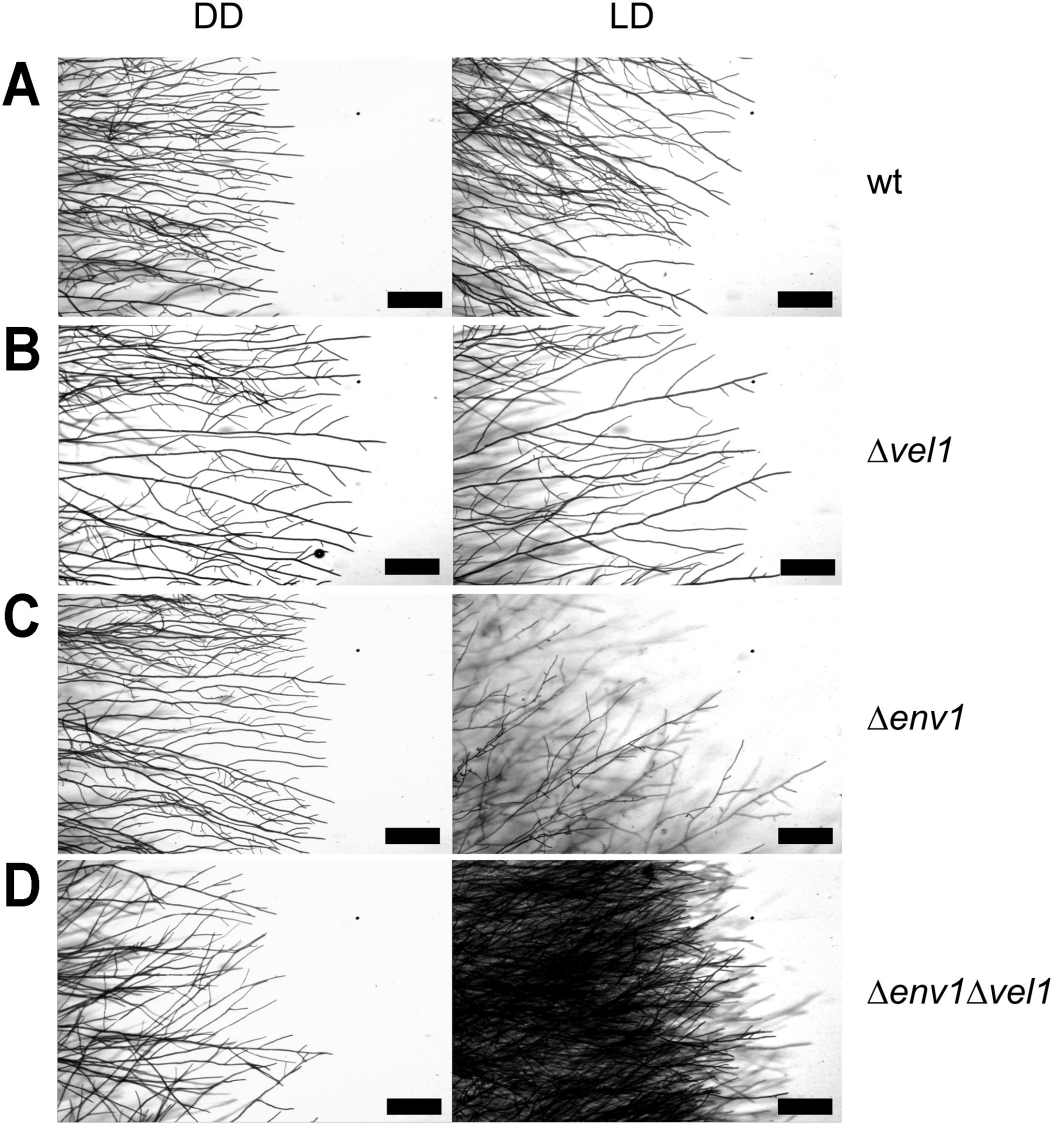
Microscopic analysis of the phenotype caused by deletion of *env1*, *vel1* or both. (A) Wildtype QM6a. (B) *Vel1* deletion strain. (C) *Env1* deletion strain as control (D) *Env1 vel1* double mutant. Strains were grown on malt extract agar (MEX) as carbon source in darkness (DD) or daylight (LD, light-dark cycles). Scale bars are equivalent to 200 μm. Lactophenol-blue staining was used to increase contrast in microscopic pictures.

Δ*env1*Δ*vel1F* double mutants did not sporulate on malt extract medium, similar to the phenotype of Δ*vel1F*. They showed a phenotype of decreased growth on malt extract medium, or minimal medium with glucose or cellulose as carbon source to around 50–60% of wildtype (Fig A in S1 File). Interestingly, the growth phenotype was somewhat alleviated in the double mutant compared to deletion of *env1* alone in light, but in darkness growth of the double mutant is generally slower than either Δ*vel1* or Δ*env1* (Fig A in S1 File). These results are in agreement with partially opposite functions of ENV1 and VEL1 in gene regulation. The growth pattern of Δ*env1*Δ*vel1F* as observed by microscopy rather showed an additive phenotype of dense mycelium (Fig 3).

**Fig 4:**
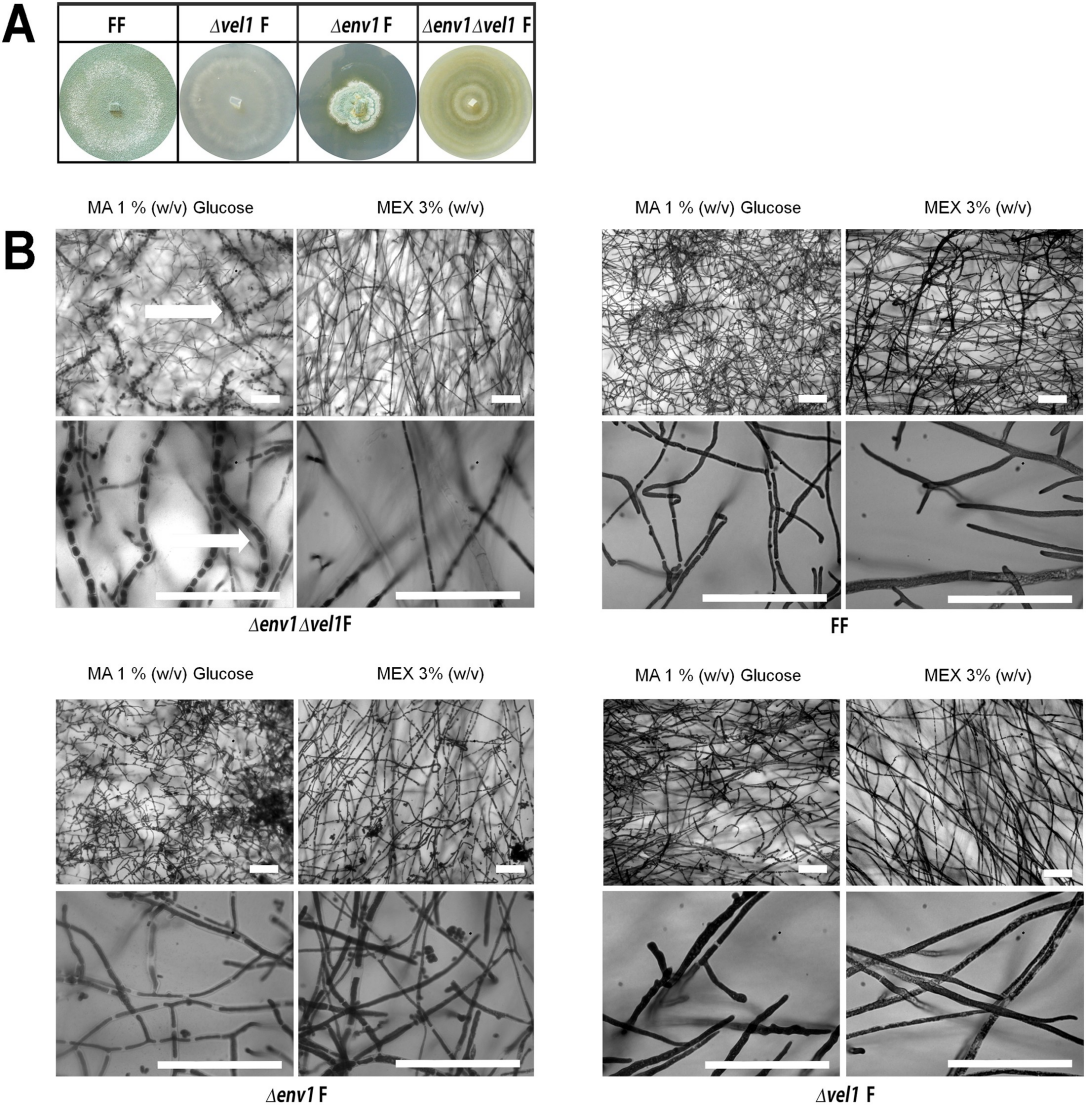
Phenotype of mutants lacking *vel1*, *env1* or both in a female fertile background. (A) *Δvel1* strains are defective in conidiation and *Δenv1* mutants in the female fertile background show a similar phenotype as described earlier in female sterile background [15] albeit with a somewhat alleviated defect in conidiation. Double mutants have a mixed phenotype compared to the single mutants. (B) Microscopic analysis using lactophenol-blue staining of *Δenv1Δvel1* double mutants and agar-block microscopy. Scale bars are equivalent to 70 μm. Strains were grown on Mandels Andreotti (MA) minimal medium with 1% (w/v) glucose as carbon source or on malt extract agar (MEX) in daylight (light-dark cycles) at 22°C for 10 days.

Around 5 days after inoculation, we further observed a greenish hue in double mutants on the plates with glucose growing in light (Fig 4A), but not on any other carbon source or in darkness. In contrast to Δ*vel1*F strains, sporulation is affected, but not abolished in strains lacking *env1* [11, 15, 16]. Hence, a partial rescue of the *vel1*-related sporulation phenotype due to deletion of *env1* seemed possible. We therefore investigated strains cultivated under the different conditions under the microscope and found structures we hypothesized to be early stages of the pathway leading to conidia formation or structures representing a partially perturbed morphological development of conidia in Δ*env1*Δ*vel1*F, without formation of conidiophores or phialides (Fig 4B, upper left panel). Alternatively, only the production of spore pigments could have been initiated without further progress to the morphological changes required for sporulation.

**Fig 5:**
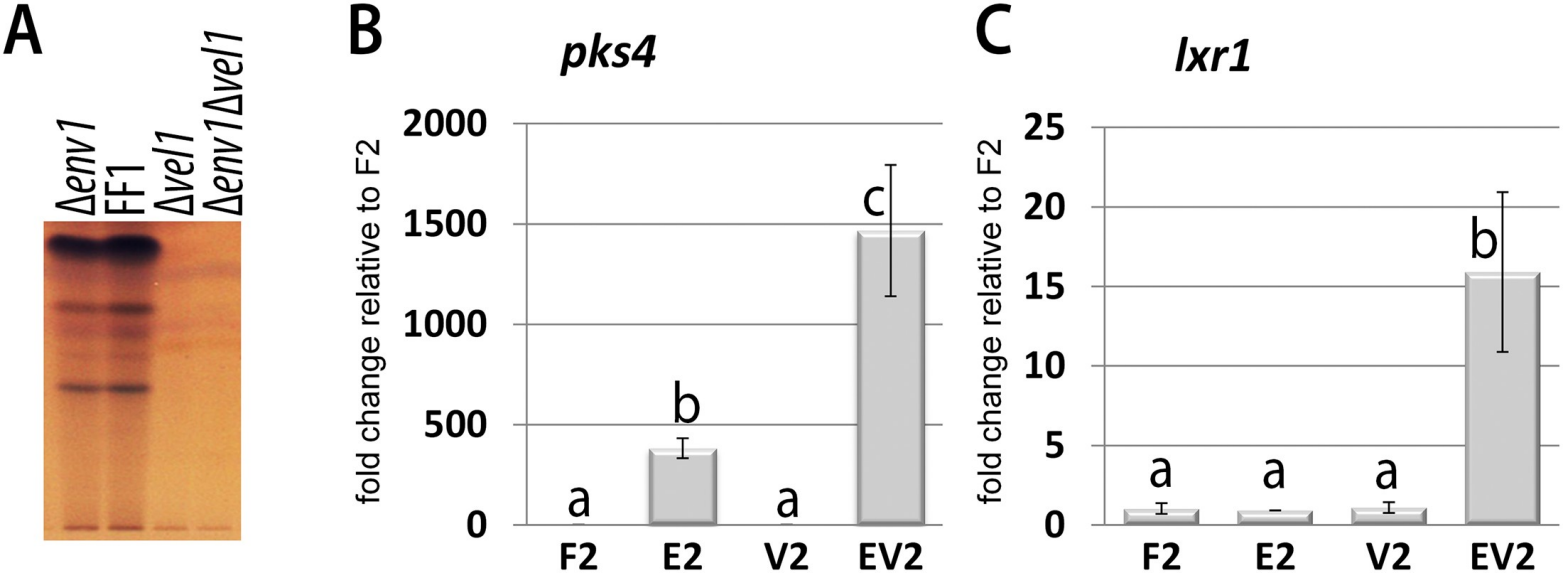
Analysis of secondary metabolite patterns and transcript levels of *pks4* and *lxr1*. (A) Secondary metabolite patterns of wildtype (QM6a) and mutant strains are shown during growth under conditions facilitating sexual development (malt extract agar, 3% w/v). Analysis was performed using high performance thin layer chromatography (HPTLC) using at least three biological replicates from pooled samples (3 cultivation plates each), which showed similar results. The figure shows the result from the derivatized HPTLC plate (transmission, visual light). Transcript abundance is shown upon growth on malt extract agar plates at subjective noon as analyzed by qRT-PCR. (B) Regulation of *pks4* or (C) *lxr1* under asexual conditions in wildtype (F2), Δ*env1F* (E2), Δ*vel1F* (V2) and Δ*env1*Δ*vel1F* (EV2). Pooled samples from several plates and at least two independent biological replicates were considered. Statistical significance of differences was evaluated with the software qbase+ and applying ANOVA analysis with a p-value threshold of p<0.05. Errorbars show standard deviations. Different letters indicate significantly different transcript levels.

We therefore evaluated the effect of the deletion of *vel1* in an *env1* negative background. As the essential activator of conidiation in other fungi, BrlA [38, 39] has no homologue in *T*. *reesei* and the conidiation pathway is not studied in detail in this fungus, we selected two genes which have been associated with conidiation previously [3, 40–42]. The obvious pigment accumulation in hyphae of the double mutant suggested an influence on conidiation associated secondary metabolite production. Therefore, we chose *pks4* (TR_82208), a polyketide synthase gene, which is responsible for the green pigmentation of *T*. *reesei* spores [40]. Due to the function of *vel1* in secondary metabolism, regulation of this gene could be expected. Global analysis of the secondary metabolite pattern of Δ*vel1*F confirmed earlier findings [34], that VEL1 is important for secondary metabolism (Fig 5A). Δ*env1* does not show a major influence on secreted metabolites and deletion of *env1* in a Δ*vel1* background did not rescue this phenotype of overall decreased secreted metabolite production (Fig 5A).

Upon growth in daylight, *pks4* was not significantly regulated by VEL1, but strongly up-regulated (more than 380fold) in the *env1*-mutant. In the double mutant, this up-regulation increased to almost 1500fold (Fig 5A), which is in agreement with a higher amount of pigment in the mycelium and hence the greenish appearance of Δ*env1*Δ*vel1F*. The second gene, *lxr1* (TR_74194) has previously been suggested as a marker gene for conidiation in *T*. *reesei* [41, 42]. Under the same conditions as for analysis of *pks4*, we found no significant regulation of *lxr1* by ENV1 or VEL1 alone. However, in the double mutant, *lxr1* was strongly upregulated (around 15fold; Fig 5B). Consequently, these results support our hypothesis that part of the pathway leading to conidiation is up-regulated in the double mutant due to the lack of ENV1. VEL1 is however essential for formation of the morphological structures required and the pathway to conidiation hence is blocked already very early after pigment formation.

### Sexual development of a Δ*env1*Δ*vel1* double mutant in light is comparable to a Δ*vel1* mutant

Single mutants of *env1* [11] and *vel1* [34] are female sterile under standard mating conditions (light—dark cycles). We were hence interested whether the double mutant would show an even more severe developmental phenotype or—as could be concluded from the results of our growth tests and conidiation effect—if the defects of single mutants are at least in part alleviated.

**Fig 6:**
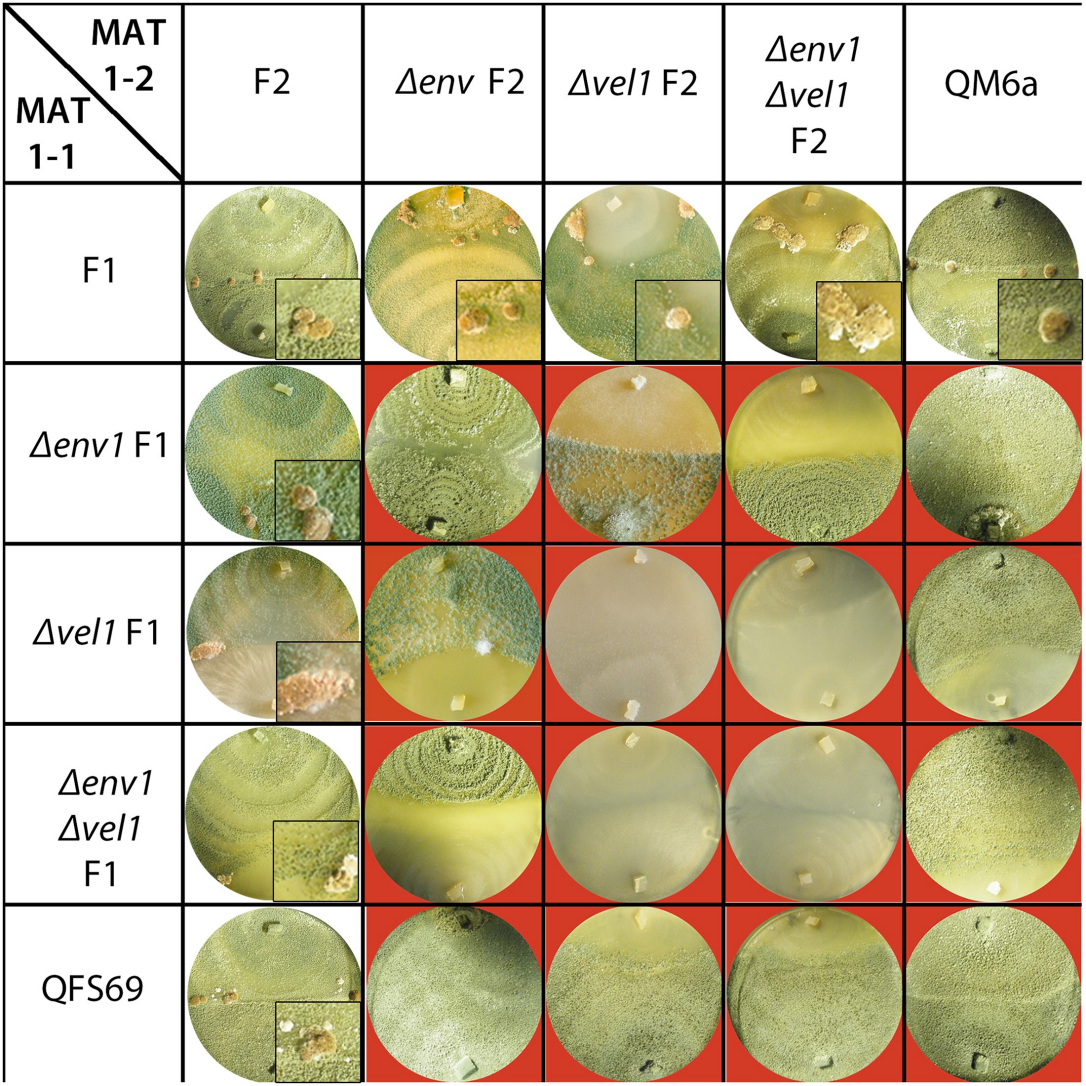
Analysis of mating and female fertility of Δ*env1*Δ*vel1F* in light. Strains were grown on malt extract agar in daylight (light-dark cycles) at 22°C for 30 days. Red background indicates no fruiting body formation and hence abolished mating.

Therefore, the double mutants were crossed with the female fertile wildtypes FF1 and FF2, the female sterile single mutants *Δenv1*F and Δ*vel1*F as well as with known female sterile test/wildtype strains (QM6a and QSF69 for mating types 1–1 and 1–2 crosses respectively) in daylight (Fig 6).

We found that female sterility as caused by deletion of *env1* or *vel1* is not alleviated in the double mutants under normal crossing conditions. The double mutants are still unable to mate with a female sterile partner or any of the single mutants (Fig 6). The wildtype strains formed fruiting bodies 7 days after inoculation. If male fertility would be perturbed, crosses of the female sterile Δ*env1*Δ*vel1*F with the wildtypes would not result in fruiting body formation, hence male fertility is not altered in these mutants. However, there was an influence of the mating type on the time point of the fruiting body appearance: Crosses of the double mutants in MAT 1–2 with the wildtype F1 resulted in fruiting bodies at the same time point of the wildtype crosses (7 days), while in crosses of the double mutants in MAT 1–1 fruiting body formation was delayed to 11 days after inoculation, in both cases with strongly decreased ascospore formation (Fig 2F). Two further repetitions under similar conditions confirmed this mating type dependent delay in fruiting body formation.

### Deletion of *env1* partially rescues fruiting body formation of Δ*vel1* in darkness

**Fig 7:**
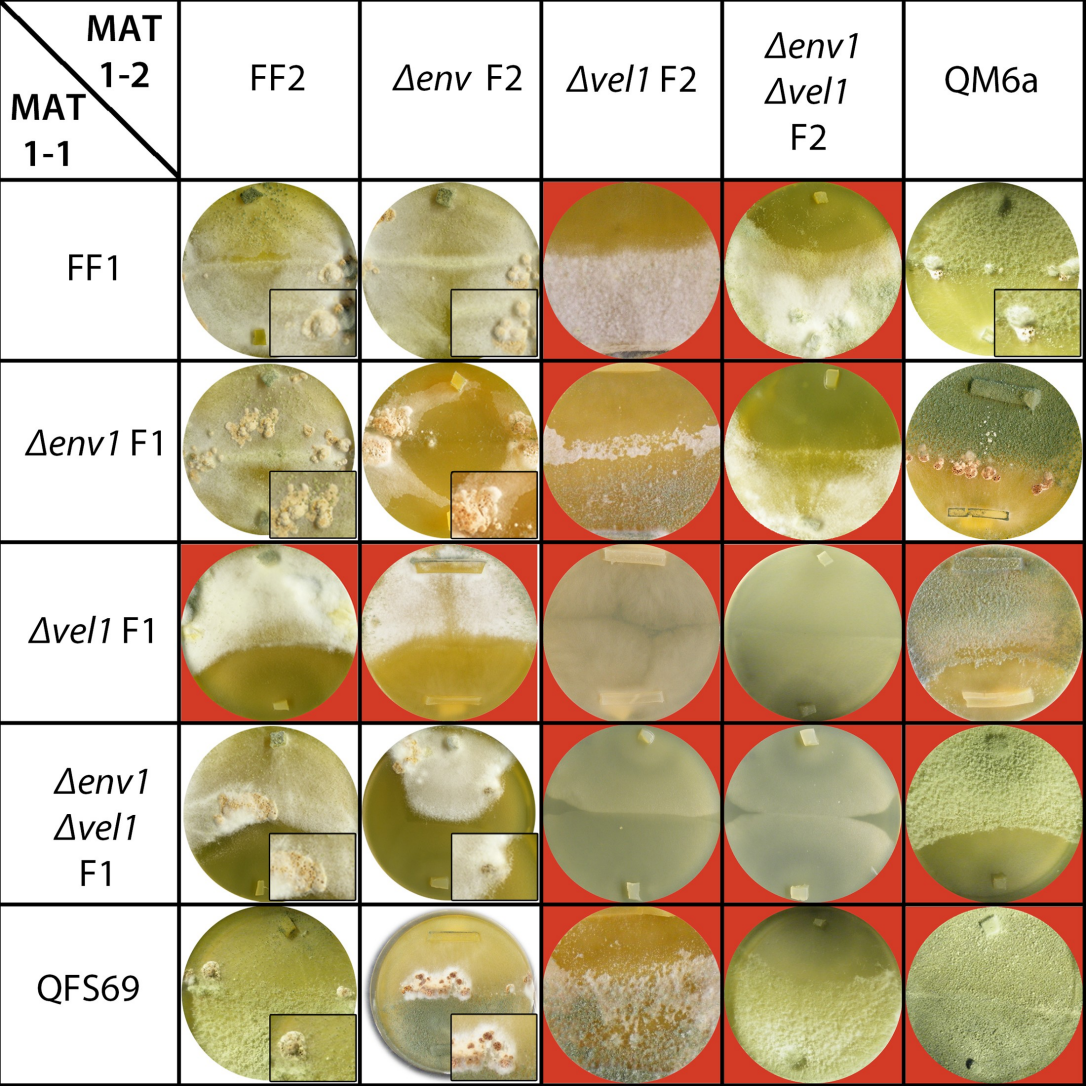
Analysis of mating and female fertility of Δ*env1*Δ*vel1F* in darkness. Strains were grown on malt extract agar in constant darkness at 22°C for 30 days. Red background indicates no fruiting body formation and hence abolished mating.

In darkness, we observed that crosses of Δ*env1*Δ*vel1*F1 with the wildtype F2 and Δ*env1*F2 resulted in fruiting bodies while similar crosses of Δ*env1*Δ*vel1*F2 did not form any fruiting bodies even after 30 days in darkness (Fig 7). Crosses of the double mutants and Δ*vel1*F were not successful irrespective of the mating type. Therefore, we conclude that the defect in sexual development of double mutants in darkness is mating type dependent and that deletion of *env1* partially rescues female fertility due to lack of *vel1* in darkness.

In summary, deletion of *env1* in *T*. *reesei* strains lacking *vel1* (no sporulation) does not result in a developmental phenotype resembling that of *vel1* mutants in *Aspergilli* (increased sporulation), [27] of which most species do not have an ENV1 homologue [32]. Our results on the light dependent influence of ENV1 on the developmental phenotype of *vel1* mutants do not indicate that the difference in light specific regulation of development is caused by the function of ENV1.

### ENV1 and VEL1 exert opposing functions in regulation of the pheromone system

In strains lacking *vel1*, effects on the mating partner present on the same plate were detected: Regulation of *hpp1* and *hpr2* was altered depending on the mating partner i. e. if *Δvel1* was the mating partner, while this effect did not occur in the wildtype [34]. On the other hand, the pheromone system of strains lacking *env1* is strongly deregulated [11]. Therefore we investigated the influence of the lack of both *env1* and *vel1* in regulation of genes involved in pheromone response.

First we re-evaluated previous data on the influence of ENV1 on the pheromone system, which indeed revealed partner effects reflected by alterations under conditions of sexual development in crosses with *Δenv1*, compared to wildtype crosses (Fig B in S1 File and [11]). While *hpp1* transcript levels decrease by roughly 50% at the contact stage, *ppg1* levels increase more than 2fold in CBS999.97 MAT1-2, if *Δenv1* is the mating partner compared to the corresponding wildtype. Additionally, ENV1 regulates *mat1-2-1* [11]. These results are in agreement with the findings described above that the regulatory interrelationship between VEL1 and ENV1 is mating type dependent.

Hence, both ENV1 and VEL1 have functions in sensing of mating partners and regulation of the pheromone system, which may result in a combined effect in double mutants: Transcript analysis of Δ*env1*Δ*vel1* showed an increase of *hpp1* transcript abundance of 1.9fold in MAT1-1 (Fig 7A; EV1). In the cognate mating type of *hpp1*, MAT1-2, the increase compared to the MAT1-2 wildtype was more than 70fold (Fig 7A; EV2). Therefore, in the double mutants transcript levels of *hpp1* do not show the strong deregulation observed in Δ*env1*, but still elevated levels compared to *vel1*-mutants [11, 34].

**Fig 8:**
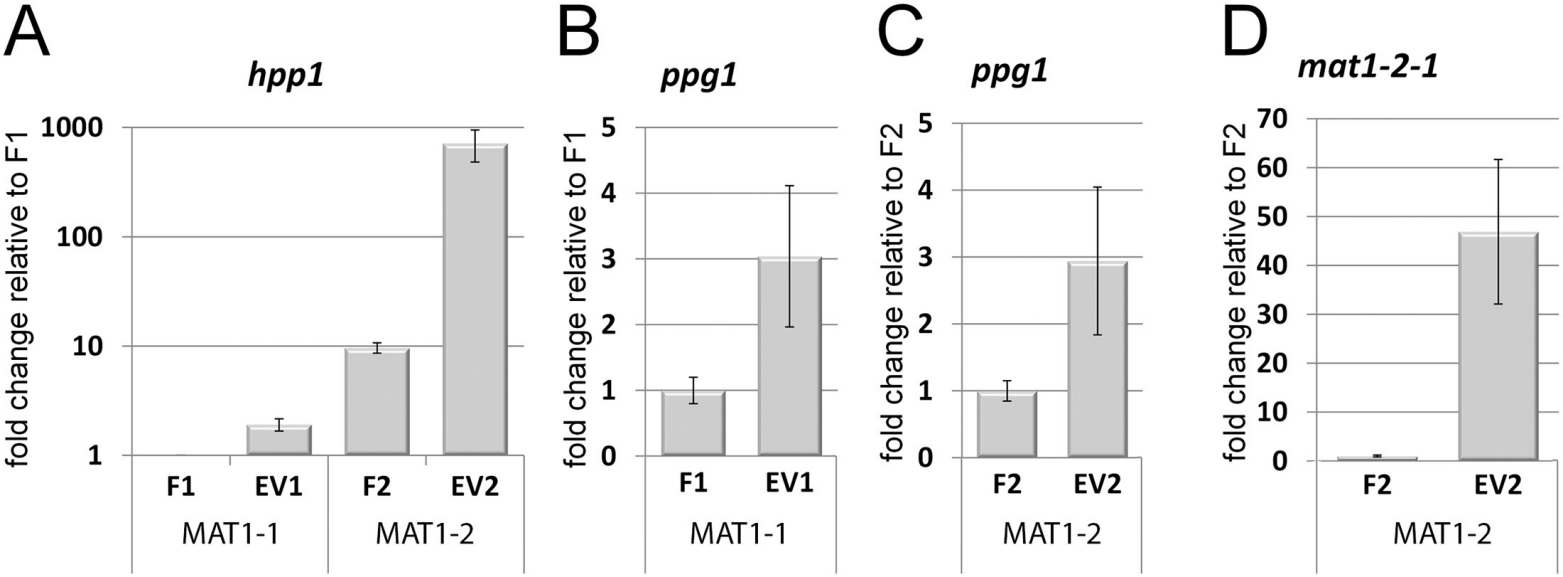
Analysis of transcript levels of pheromone system genes in double mutants. Transcript abundance is shown upon growth on malt extract agar plates at subjective noon as analyzed by qRT-PCR. (A) Regulation of *hpp1* or (B, C) *ppg1* or *mat1-2-1* (D) under asexual conditions in wildtype (F1, F2) and Δ*env1*Δ*vel1F* (EV1, EV2). Results for *ppg1* are separated into two panels for better comparison. Transcript levels in F1 are 65±11fold higher than in F2. Pooled samples from several plates and at least two independent biological replicates were considered. Statistical significance of differences was evaluated with the software qbase+ and applying ANOVA analysis with a p-value threshold of p<0.05. Errorbars show standard deviations.

For *ppg1*, the increase in transcription levels in the double mutant was roughly 3fold compared to wildtype consistently in both mating types, with the expectedly higher transcript levels of *ppg1* MAT1-1 versus MAT1-2 (65±11fold; Fig 8B and 8C). *ppg1* shows only moderate upregulation in the double mutant, resembling regulation in Δ*vel1*, despite the strong regulation reported for Δ*env1*.

For the mating type gene *mat1-2-1*, we detected 46fold up-regulation in the double mutant.

Consequently, the function of VEL1 interferes with the pathway deregulated in Δ*env1*, that is responsible for deregulation of the pheromone system. Nevertheless, pheromone expression must involve additional regulators besides VEL1, that cause the residual strong up-regulation of *hpp1* and the moderate up-regulation of *ppg1* in the double mutant. Hence ENV1 and VEL1 act like checkpoints in the regulatory cascade, with the deregulation caused by lack of *env1*. The function of VEL1 appears moreover to also contribute to the strongly increased pheromone levels in Δ*env1*.

In contrast, VEL1 is not involved in up-regulation of mat*1-2-1* in Δ*env1* [11], because the strongly elevated levels in the double mutant match those in Δ*env1*. This result is in agreement with earlier findings, that VEL1 does not influence *mat1-2-1* transcript levels [34].

## Discussion

**Fig 9:**
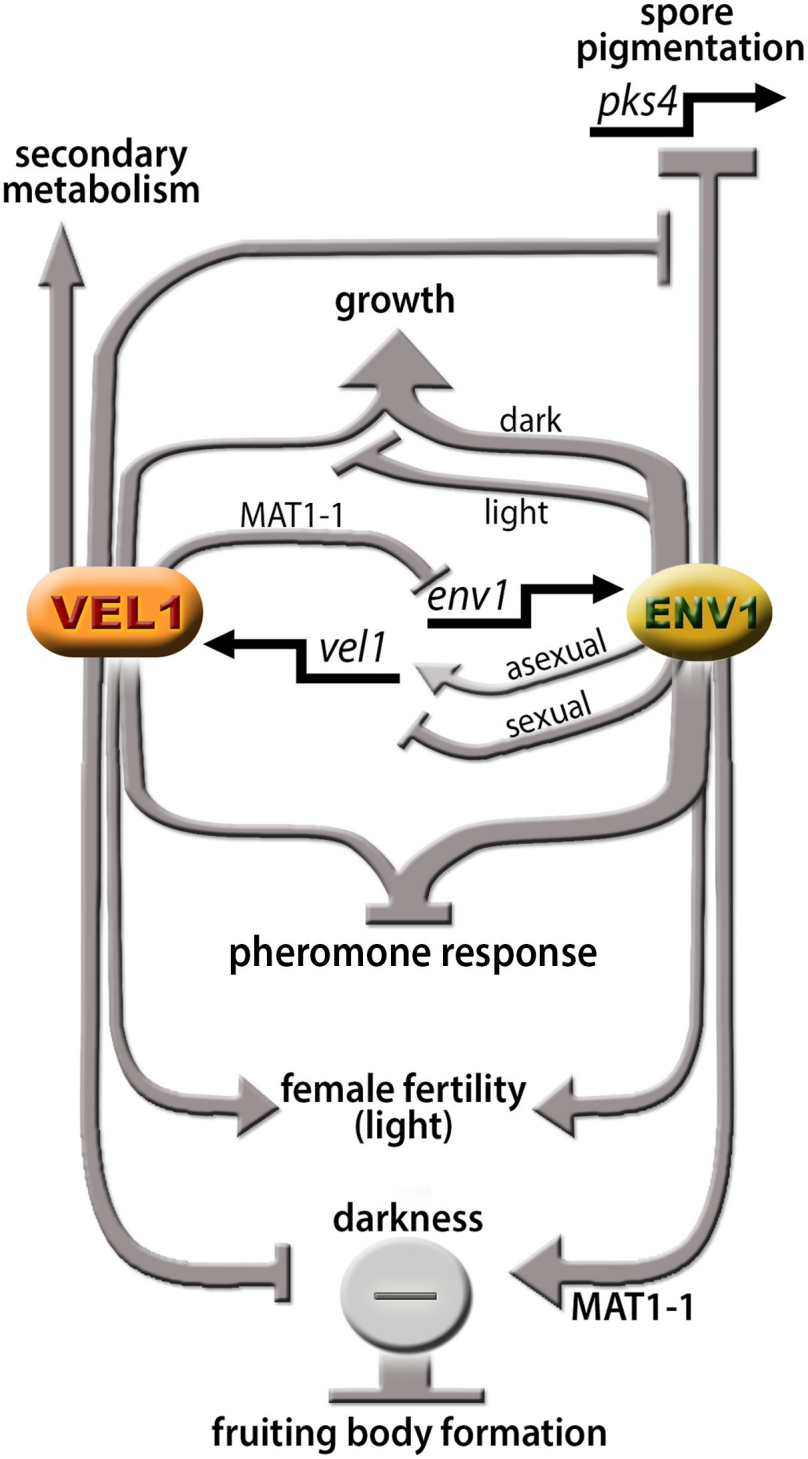
Model for the functions and interrelationships of ENV1 and VEL1. ENV1 and VEL1 show a mutual regulatory interaction at the level of transcription. Both genes contribute to regulation of pheromone response. ENV1 and VEL1 are essential for female fertility in light. In darkness, regulation of fruiting body formation is balanced by the effect of ENV1 and VEL1 on a negative regulator in MAT1-1. If only VEL1 is lacking, the positive effect of ENV1 on the repressor leads to a block of fruiting body formation. Deletion of ENV1 abolishes this positive effect and alleviates the block, which is also in agreement with the only minor function of BLR1 and BLR2 (responsible for induction of *env1*) in darkness. The largely positive function of VEL1 on secondary metabolism is independent of ENV1, although the specifically negative effect of ENV1 on transcription of *pks4* and hence spore/mycelium pigmentation is decreased by VEL1. The effect of ENV1 and VEL1 on growth is light dependent. Arrows indicate a positive function, plungers show a negative effect.

Sexual development is an outstanding achievement through evolution which results in increased genetic variation and improved fitness of organisms under harsh environmental conditions [43]. Molecular pathways involved in regulation of development have been extensively studied in fungi and in this respect, light was shown to play an important role [9, 44]. Accordingly, light and the light signaling machinery were found to have important functions in regulation of sexual development in *T*. *reesei* [5, 11]. Previously, we showed that VEL1, the homologue of *A*. *nidulans* VeA in *T*. *reesei*, is involved in secondary metabolism and light-dependent regulation of sexual development [34]. Identifying both ENV1 and VEL1 to be important in sexual development, we intended to gain insight into the interrelationship of these two major components in this pathway (Fig 9).

We observed that in a Δ*env1Δvel1* mutant the ability to mate with the wildtype (FF2) and Δ*env1* in darkness is restored (Fig 7), although transcriptional regulation of the pheromone system in Δ*env1* in darkness shows only minor alterations compared to wildtype [11]. Hence we consider it unlikely that the restored mating ability of the double mutant is due to an altered pheromone regulation by deletion of *env1*. Rather, we propose that the function of ENV1 in darkness is not independent from VEL1. We suggest that at least in MAT1-1, ENV1 and VEL1 balance the function of a negative regulator of fruiting body formation in darkness (Fig 9). In the absence of VEL1, the pathway triggered by ENV1 appears to block sexual development in darkness and this block is alleviated if *env1* is deleted as well. Interestingly, this effect is mating type dependent and only occurs in MAT1-1. This dependency is in agreement with a different effect of ENV1 on the pheromone system depending on mating type as shown previously [11]. Additionally, deletion of the photoreceptors, which positively regulate *env1* transcript levels, does not have a major influence on fruiting body formation in darkness [11], which is in line with this interpretation as well.

We conclude that ENV1 is likely to have a mating type dependent negative effect on sexual development in darkness, which is counteracted by VEL1 in the wild-type. In the absence of VEL1, mating would then be blocked due to this negative effect of ENV1. Another, yet unknown factor is likely to fulfil this negative function in MAT1-2 and hence the block is not alleviated in this mating type (Fig 9). It will be interesting to learn, which light- and mating type dependent components are responsible for the output of the balanced regulation of sexual development by ENV1 and VEL1.

ENV1 has important functions in light dependent gene regulation [20, 45] and is essential for female fertility in light [11]. We observed regulation of *vel1* by ENV1, which was increased upon encounter of a potential mating partner, and *env1* was regulated by VEL1 in a mating type dependent manner. Also, the patterns in sexual and asexual development differed (Fig 9). Thus, ENV1 is proposed to act as an additional checkpoint for signal integration with VEL1 rather than strictly sequentially in the signaling cascade. Interestingly, we found that the defect in female fertility, which was observed in both strains, is condition dependent. At least in the strain lacking ENV1, female fertility due to deregulation of the pheromone system [11] can be overcome with altered light exposure times. In some cases our results indicated that lack of ENV1 partially alleviates the defects of Δ*vel1F* strains or vice versa, as in several cases the phenotype of the double mutant is between the two single mutants. In contrast, with respect to regulation of conidiation associated genes (*lxr1* and *pks4*) an unexpectedly strong effect was found for the double mutant, which considerably exceeds the regulation by single mutants, reflecting an effect of ENV1, which is negatively influenced by VEL1 (Fig 9). Deletion of *vel1* therefore even increases the positive effect of lacking *env1* on *pks4* transcription in the single mutant.

We interpret this phenomenon as an indication that ENV1 and VEL1 both play important and complementing roles in conidiation specific gene regulation, albeit at a very early step. However, our results do not suggest direct regulation, but effects on positive and negative regulators. These regulators are kept in balance by the functions of ENV1 and VEL1. While lack of ENV1 releases part of the block, the negative effect of VEL1 can only be alleviated in the absence of ENV1. This interpretation would be in agreement with a double lock mechanism. A comparable mechanism is suggested for sexual development as outlined above. This effect is mating type dependent, as are other effect previously shown for ENV1 [11]. It will be interesting to learn, which factor fulfils this task in MAT1-2 as well as which regulators—for example those that are assuming the function of BrlA in *T*. *reesei*—are balanced by ENV1 and VEL1.

As *Aspergilli* have no ENV1 homologue [32], its function in regulation of VEL1 and VEL1-related pathways becomes even more interesting. Studying the interrelationship between VEL1 and ENV1 we also aimed to explain important differences in light dependent development, that are triggered by ENV1 and its homologues. From our findings we cannot conclude that the function of ENV1 complements that of VEL1 to achieve the same effect as VeA in *A*. *nidulans*. This hypothesis is further supported by the data on deletion of *ve-1* in *N*. *crassa*, which in contrast to the situation in *T*. *reesei* causes increased conidiation [46], although *N*. *crassa* has an ENV1 homologue (VVD; [17, 47]). Nevertheless, a contribution of ENV1 in *T*. *reesei* to effects caused by VeA alone in *A*. *nidulans* cannot be excluded.

Our investigation of the interrelationship of regulation of ENV1 and VEL1 revealed that their regulatory function is interconnected in balanced regulation of positive and negative output factors. In agreement with light dependent functions for both ENV1 and VEL1, we found that their interrelationship is influenced by light and in part depends on the mating type, which may in part be due to the involvement of ENV1, but not VEL1 in regulation of the mating type gene *mat1-2-1*. The different phenotypes of Aspergillus *veA* mutants compared to the double mutant Δ*env1*Δ*vel1* indicates further that the different light dependent developmental phenotypes of strains lacking VeA/VEL1 in *Aspergilli* and *T*. *reesei* are not due to the presence or absence of ENV1.

## Materials and methods

### Microbial strains and culture conditions

*T*. *reesei* (*H*. *jecorina*) QM6a wildtype strain (ATCC 13631), QFS69 (MAT1-1, female sterile derivative of QM6a), FF1 and FF2 (female fertile derivatives of QM6a), CBS999.97 MAT1-1 and MAT1-2 strains were used in this study (Table 1).

**Table 1:** Strains used in this study.

Strain	Code	Characteristics	Source/Reference
QM6a	QM	Wildtype MAT1-2	[52]
CBS999.97 MAT 1–1		Wildtype MAT 1–1	[5]
CBS999.97 MAT 1–2		Wildtype MAT 1–2	[5]
CBS999.97 MAT 1–1 Δ*hpr1*		Δ*hpr1*::hph+ MAT1-1	[7]
CBS999.97 MAT 1–2 Δ*hpr1*		Δ*hpr1*::hph+ MAT1-2	[7]
FF1	F1	Female fertile derivative of QM6a MAT 1–1	[34]
FF2	F2	Female fertile derivative of QM6a MAT 1–2	[34]
QFS69 MAT 1–1	FS	Female sterile derivative of QM6a MAT 1–1	[34]
QM6a Δ*vel1*		Δ*vel1*::*amds*^+^ MAT 1–2, female sterile background (QM6a)	[34]
QM6a Δ*env1*		Δ*env1*::*hph*^+^ MAT 1–2, female sterile background (QM6a)	This study
Δ*vel1*F1	V1	Δ*vel1*::*amds*^+^ MAT 1–1, female fertile background	[34]
Δ*vel1*F2	V2	Δ*vel1*::*amds*^+^ MAT 1–2, female fertile background	[34]
Δ*env1*F1	E1	Δ*env1*::*hph*^+^ MAT 1–1, female fertile background	This study
Δ*env1*F2	E2	Δ*env1*::*hph*^+^ MAT 1–2, female fertile background	This study
Δ*env1*Δ*vel1*F1	EV1	Δ*env1*Δ*vel1*::*hph*^+^::*amdS*^+^ MAT 1–1, female fertile background	This study
Δ*env1*Δ*vel1*F2	EV2	Δ*env1*Δ*vel1*::*hph*^+^::*amdS*^+^ MAT 1–2, female fertile background	This study

Strains were propagated on 3% (w/v) malt extract-agar (Merck, Darmstadt, Germany). Standard crossing conditions are 2% (w/v) malt extract-agar at 22°C in daylight (cycles of 12h light-12h dark). For crosses in constant darkness the same conditions were applied except that light-tight boxes were used for cultivation. For crossing, strains were grown on opposite sides of petri dishes. Fruiting body formation and ascospore discharge were checked 7 and 20 days after inoculation or as noted with the experiments. Crosses were repeated at least twice. For analysis of growth and conidiation, MA (Mandels-Andreotti) medium [48] supplemented with 1% (w/v) carbon source and 0.1% (w/v) peptone (chemicals from Merck, Darmstadt, Germany) to induce germination was used.

Precultures for transcriptional analysis were done on 3% (w/v) malt extract agar in constant darkness for at least three days. Strains were then inoculated on 2% (w/v) malt extract agar plates covered with cellophane to facilitate harvesting. Mycelia were harvested at subjective noon close to the contact zone at the stage of contact as shown in [34]; Fig 3 there). Samples from 5 plates were pooled and at least two biological replicates were used for qRT-PCR analysis. Mycelia from asexual growth were harvested from strains grown alone on plates under otherwise equal conditions. The strains were grown under cycles of 12h light-12h darkness at 22°C (1800 lux).

Cellulase analysis was done using plate assays with 1% (w/v) CMC (Sigma Aldrich, St. Louis, USA) as carbon source as described previously [34, 49].

### Nucleic acid isolation and transcript analysis

Total RNA was isolated using the RNeasy plant mini kit (QIAGEN, Hilden, Germany) as described previously [45]. Crossing partner contamination for samples under conditions of sexual development was analyzed by the relative abundance of mating type genes in co-precipitated chromosomal DNA in the RNA samples [11]. We only found negligible amounts of cross contamination by the other mating type on the plate. RNA quality control was performed agarose gel electrophoresis and staining with SYBR^®^ Safe DNA (Invitrogen, Carlsbad, USA) and the Agilent 2100 Bioanalyzer platform using RNA 6000 Nano Kit (Agilent, Santa Clara, USA). Concentration was determined by the Nanodrop ND-1000 spectrophotometer (PEQLAB, Erlangen, Germany). Only samples with a RIN (RNA integrity number) > 9 were used for further analysis [50]. DNase I treatment, cDNA analysis and qRT PCR were performed as described previously [34, 45]. Sequences of gene specific primers used in this study are provided in S1 Table in S1 File. *rpl6e* encoding the ribosomal protein RPL6e was used as reference gene. Cycle threshold (CT) values were determined for a minimum of two biological replicates and three technical replicates. All data were analyzed with the qbase+ software package (Biogazelle, Gent, Belgium) [51]. Statistical analysis of the qPCR data was performed with the qbase + (ANOVA, Tukey-Kramer post-hoc-test, p<0.05).

### Microscopy

For microscopy, strains were grown on 3% (w/v) malt extract agar in daylight (12:12 cycles) and constant darkness at 22°C. Mycelia grown on agar blocks were stained with a drop of lactophenol blue stain (Sigma, St. Louis, US). Samples were examined under a Nikon Eclipse E200 light microscope (Nikon, Tokio, Japan).

### Analysis of secondary metabolites

For secondary metabolite analysis by high performance thin layer chromatography (HPTLC), strains were grown on agar plates with malt extract medium. HPTLC was done as described previously [34], except that chloroform and 1 mM trifluoroacetic acid in methanol was used for separation. Three biological replicates were considered and for each replicate, three cultivation plates were pooled.

## Supporting information

S1 File This file contains Figure A, showing hhyphal extension of mutants in ENV1 or VEL1, Figure B, which shows partner effects in a cross of Δ*env1* with WT and Table A providing an overview on oligonucleotides used in this study. (PDF)
