# Supplementary material for: Interrelationships of VEL1 and ENV1 in light response and development in Trichoderma reesei
Source: PLoS One. 2017 Apr 19;12(4):e0175946. doi: 10.1371/journal.pone.0175946 (PMC5397039; doi:10.1371/journal.pone.0175946)
Supplement: S1 File — (PDF) [file pone.0175946.s001.pdf]

# Interrelationships of VEL1 and ENV1 in light response and development in *Trichoderma reesei*

Hoda Bazafkan, Christoph Dattenböck, Eva Stappler, Sabrina Beier and Monika Schmoll

# SUPPLEMENTARY MATERIAL

Figure A

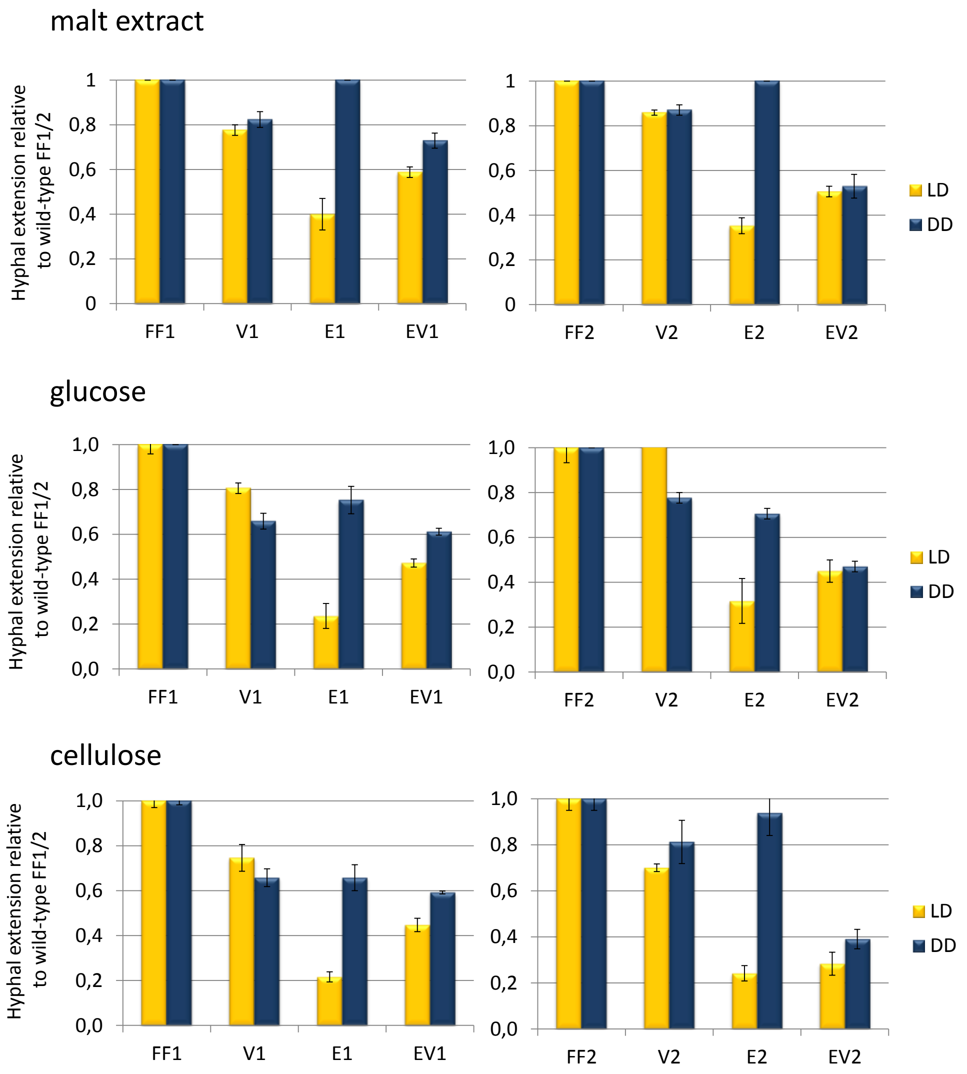


**Fig A. Hyphal extension of mutants in ENV1 or VEL1**. Hyphal extension was measured after 3 days of growth at 28°C on malt extract medium or Mandels Andreotti Minimal medium with glucose or cellulose (carboxymethylcellulose) as carbon source in constant darkness (DD) or daylight (LD; light –dark cycles).

FF1 (MAT1-1) and FF2 (MAT1-2), female fertile wild-type strains derived from QM6a [1]; V1 (MAT1-1) and V2 (MAT1-2), deletion strains of *vel1* in the female fertile background of FF1 or FF2; E1 (MAT1-1) and E2 (MAT1-2), deletion strains of *env1* in the female fertile background of FF1 or FF2; EV1 (MAT1-1) and EV2 (MAT1-2), double deletion strains of *vel1* and *env1* in the female fertile background of FF1 or FF2.

Figure B

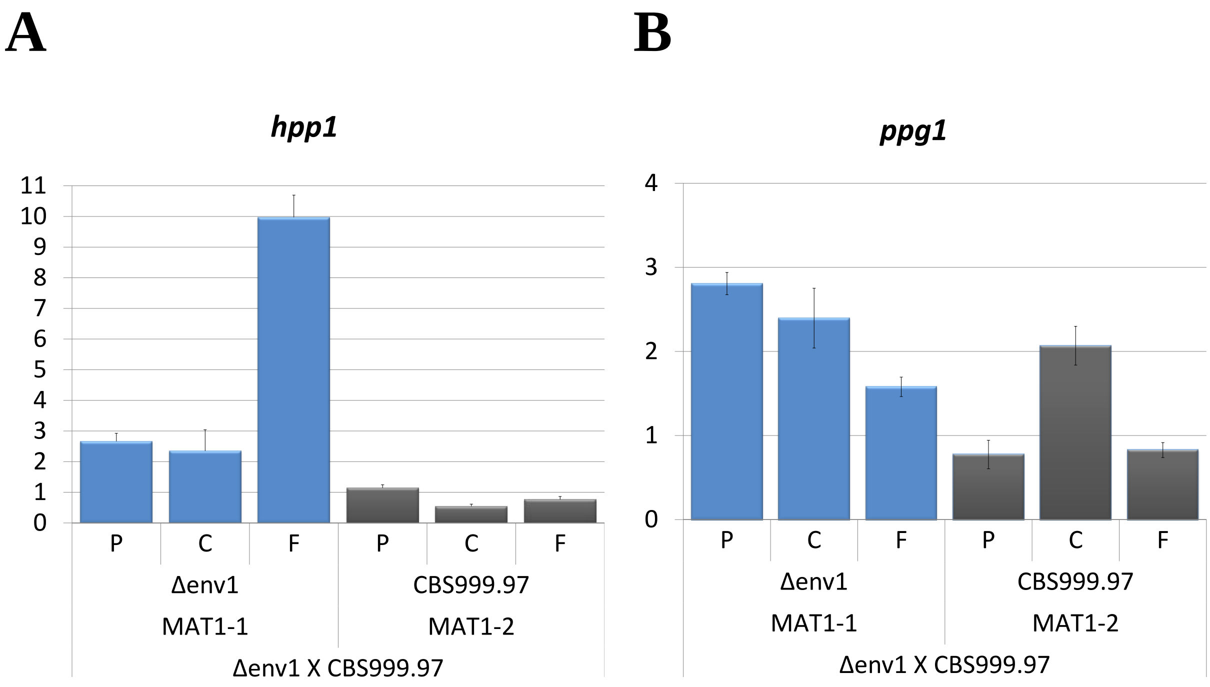


**Fig B. Partner effects in a cross of Δ*env1* with WT.** Transcript levels as determined by RT-qPCR of the peptide pheromone precursors *hpp1* (A) and *ppg1* (B) upon confrontation of CBS999.97 MAT1-2 with Δ*env1* MAT1-1 relative to a cross with wild-type. Strains were grown on malt extract agar in 12:12 light-dark cycles for initiation of sexual development. Mycelia were harvested before contact (P), upon contact (C) and at the stage of onset of fruiting body formation (F). Re-evaluation of data reported in [2].

**Table S1 Oligonucleotides used in this study**

| Oligo-nucleotide | Sequence | Purpose | Reference |
|---|---|---|---|
| ENVScreen_F1 | 5′ ACTGCGTGTTACCAAATG 3′ | Screening deletion of *env1* | [1] |
| ENVScreen_R1 | 5′ CAACATGAACCTGGAAGC 3′ | Screening deletion of *env1* | [1] |
| ppg1_c_qF | 5′ CCGTCCTGAGCGCCACCATT 3′ | qRT-PCR *ppg1* | [1] |
| ppg1_c_qR | 5′ CGATGACGCTGCGAGCAACG 3′ | qRT-PCR *ppg1* | [1] |
| hpr1_c_qF | 5′ ATCCGCTTCCGCCAAGTCAC 3′ | qRT-PCR *hpr1* | [1] |
| hpr1_c_qR | 5′ CAGGGGGTGGACGAGGATGA 3′ | qRT-PCR *hpr1* | [1] |
| RT_VEL_F1 | 5′ CGAGGAGGGCAAGGACATTAC 3′ | qRT-PCR *vel1* | [1] |
| RT_VEL_R1 | 5′ GCAGGAACACCAGTCAGGATG 3′ | qRT-PCR *vel1* | [1] |
| RT_env1F | 5′ GCCCTCTCGACTGCTCCGTC 3′ | qRT-PCR *env1* | [3] |
| RT_env1R | 5′ CGACCCATGATCTCGGGGGC 3′ | qRT-PCR *env1* | [3] |
| hpp1F | 5′ ACAATCACCGTGGGACATCTG 3′ | qRT-PCR *hpp1* | [4] |
| hpp1R | 5′ TCCCTGCTGTTCCGCTGATG 3′ | qRT-PCR *hpp1* | [4] |
| hpr2F | 5′ TGGCACCACTTCATCAACTTC 3′ | qRT-PCR *hpr2* | [4] |
| hpr2R | 5′ GGAGTAGGAGGAGGATGTGTTG 3′ | qRT-PCR *hpr2* | [4] |
| RT_74194_F | 5‘ TTGGGACCATGTCATCCAGGTC 3‘ | qRT-PCR *lxr1* | This study |
| RT_74194_R | 5‘ ATAACCGGGCGAAATGCTGTTG 3‘ | qRT-PCR *lxr1* | This study |
| RT_82208_F | 5‘ ACTGAAGCAGTATCGGGCAACT 3‘ | qRT-PCR *pks4* | This study |
| RT_82208_R | 5‘ TCTTCGACGTAAAGAGCAGCCA 3‘ | qRT-PCR *pks4* | This study |

## References


1. Bazafkan H, Dattenböck C, Böhmdorfer S, Tisch D, Stappler E, Schmoll M: **Mating type dependent partner sensing as mediated by VEL1 in *Trichoderma reesei***. *Mol Microbiol* 2015, **96**(6):1103-1118.

2. Seibel C, Tisch D, Kubicek CP, Schmoll M: **ENVOY is a major determinant in regulation of sexual development in *Hypocrea jecorina* (*Trichoderma reesei*)**. *Eukaryot Cell* 2012, **11**:885-890.

3. Tisch D, Kubicek CP, Schmoll M: **New insights into the mechanism of light modulated signaling by heterotrimeric G-proteins: ENVOY acts on *gna1* and *gna3* and adjusts cAMP levels in *Trichoderma reesei* (*Hypocrea jecorina*)**. *Fungal Genet Biol* 2011, **48**(6):631-640.

4. Seibel C, Tisch D, Kubicek CP, Schmoll M: **The role of pheromone receptors for communication and mating in *Hypocrea jecorina* (*Trichoderma reesei*)**. *Fungal Genet Biol* 2012, **49**(10):814-824.
